# *dl*‐Limonene as an Alternative Sustainable Solvent to Xylenes for the Industrial Preparation of Release Formulations

**DOI:** 10.1002/open.202400259

**Published:** 2024-12-10

**Authors:** Giuseppe D'Orazio, Rosa Ragone, Antonino Rizzuti, Francesca Serena Abatematteo, Alessandra Ciampa, Giuseppe Ghisa, Michela Casini, Mario Latronico, Vito Gallo, Piero Mastrorilli, Biagia Musio

**Affiliations:** ^1^ Department of Civil, Environmental, Land, Building and Chemical Engineering, DICATECh Polytechnic University of Bari Via Edoardo Orabona 4 70125 Bari Italy; ^2^ Department of Chemistry University of Milan via C. Golgi 19 Milano 20133 Italy; ^3^ IFOM ETS - The AIRC Institute of Molecular Oncology via Adamello 16 Milano 20139 Italy; ^4^ Istituto Poligrafico e Zecca dello Stato via del Mare 32 Foggia 71121 Italy; ^5^ Istituto Poligrafico e Zecca dello Stato via Salaria 691 Roma 00138 Italy

**Keywords:** Peelable coating, Bio-wax, Renewable chemical, Multilayer foil, Release agent, Green solvents

## Abstract

Many industrial processes use aromatic hydrocarbons as solvents, including benzene, toluene, ethylbenzene, and xylene (BTEX). However, their use is discouraged due to their toxicological profile. Research is ongoing to find alternative more sustainable solvents. This work explores the adoption of *dl*‐limonene as an alternative to BTEX for the industrial preparation of release formulations containing carnauba wax to be employed in the peelable foils industry. A preliminary chemical‐physical characterization of carnauba wax was carried out using spectroscopic (ATR‐FTIR, NMR) and thermal analyses (DSC). Based on the chemical composition found for the carnauba wax used in the present study, different solvent mixtures and different concentrations of carnauba wax were tested to obtain a clear and stable solution. The most promising formulations were subjected to a coating test on a solid substrate (polyethylene terephthalate, PET) on a laboratory scale, followed by a peeling test to assess the resulting peeling strength. The *dl*‐limonene/ethyl acetate mixture in the 9 : 1 ratio has been identified as the most suitable to completely solubilize carnauba wax (0.38 wt/vol %) and obtain an adequate release performance. The results of these experiments were then used to set up and successfully perform an industrial‐scale coating test.

## Graphical abstract

Aromatic hydrocarbons are widely used solvents in chemical processes. Due to concerns about toxicity to the environment and human health, the introduction of more sustainable alternative solvents is highly desirable. In this work, the use of *dl*‐limonene was explored to develop formulations containing carnauba wax as a release agent in the production of peelable foils.

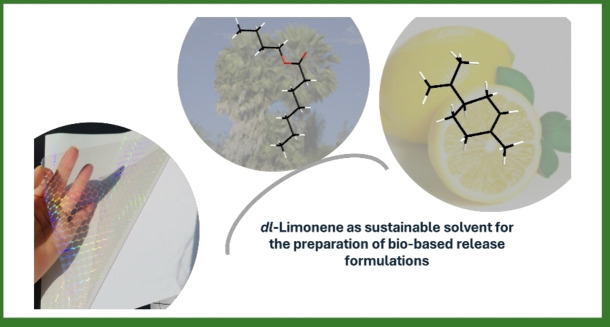

## Introduction

Adopting a more sustainable use of solvents during chemical processes has become an urgent issue considering the impact these substances have on human health and the environment.[^1^, ^2^, ^3^, ^4^, ^5^] The impact of these substances is made even more serious by the fact that during a chemical process, the fraction of solvent compared to that of the other chemical reagents is generally very high. In such circumstances, a large amount of waste is generated.[^6^, ^7^, ^8^] When developing an industrial chemical process, it is necessary to carry out an accurate selection of the solvents considering the following aspects: waste production and management (incineration, recycling, biotreatment, and emissions of volatile organic compounds),^[9]^ environmental impact, health hazard, and safety (flammability, explosion, reactivity, and stability).^[10]^ Nowadays, the introduction of enabling technologies helped to reduce significantly the fraction of solvent employed during a range of chemical processes and to enhance its recycling.[^11^, ^12^, ^13^] However, it should be considered that many complexities are associated with the chemical composition of the waste stream, including the technoeconomic and environmental aspects, which make the recycling process very difficult.^[14]^ Many solvent guides based on life cycle analysis assessment (LCA) and environmental, safety, and health (ESH) classification have been published so far.[^15^, ^16^, ^17^] However, understanding the physicochemical properties of both the solvent and the substance to be solubilized remains one of the most crucial parts of developing a sustainable process with minimization of environmental and toxicological impact.

In the present work, these aspects were taken into consideration during a specific industrial case study, which involves the production of peelable films intended for the lamination sector.

**Figure 1:**
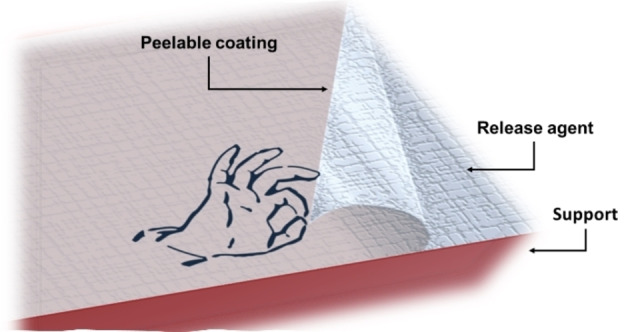
Schematic picture of the function of a release agent during the removal of a peelable foil.

The application area of the peelable coating is extremely wide, including anti‐corrosion coatings, food casing, decontamination, automotive and floor coatings, and peelable labeling.^[18]^ An important feature of peelable films is their ability to be stripped off from a surface uniformly and with ease, with no loss of fragments, as illustrated in Figure 1.

To achieve such results, molding releasing agents are very often employed and incorporated into the multi‐layer structure of the peelable film. A mold release agent should ideally have the following properties: be non‐toxic and safe to use; be quick and easy to apply; form a complete and uniform film on the mold surface; have a low coefficient of friction to facilitate easy release; do not give transfer to the printed surface; have a low cost.^[19]^ Unfortunately, none of the chemicals nowadays available as molding release agents have all these properties simultaneously. However, waxes, such as bees and carnauba waxes, are characterized by many of the aforementioned physicochemical properties which make them particularly suitable as a molding release agent in industrial processes. Carnauba wax, also known as Brazil wax or ceara wax, belongs to this class of substances and finds very diverse applications as food‐grade polish, hardening or gelling agent, and a component of polishes in furniture, leather, car, and shoe industries. It is also used as an additive for medicines, cosmetics, cleaning products, plastics, films, as a thickener for solvents and oils, and as a hardener for printing inks.[^20^, ^21^] Carnauba wax is obtained from both the young and more mature leaves of the carnauba palm (Copernicia prunifera) of Brazil. Brazilian technical regulations define the wax powder coming from the two types of leaves as A and B, respectively.[^22^, ^23^] The specific behavior of carnauba wax in dependence on temperature variations makes it particularly suitable as a molding release agent.[^24^, ^25^, ^26^] One of the limitations in the application of this substance as a coating agent is its poor solubility in traditional solvents employed on an industrial scale, being insoluble in water, partially soluble in boiling alcohol and non‐polar solvents, soluble in ether, chloroform, and oil.[^27^, ^28^]

In the context of peelable film production, during the coating stage, carnauba wax is generally solubilized in a mixture of heated aromatic hydrocarbons, such as toluene and xylene. Benzene, toluene, ethylbenzene, and xylene, often referred to as BTEX, belong to the class of aromatic hydrocarbons (AHs) and are of particular concern for human health, being reported as neurotoxins and irritants.^[29]^ The International Agency for Cancer Research (IARC) classified benzene and ethyl benzene as carcinogenic compounds in humans and experimental animals, respectively.[^30^, ^31^] Although xylene and toluene are not classified as carcinogens,[^32^, ^33^] many studies have been reported on their toxicological effects following inhalational, oral, dermal, and ocular exposure. Among BTEXs, xylene represents one of the most studied VOCs, being particularly prone to undergoing oxidative transformations with the formation of by‐products potentially dangerous for the environment and human health.[^34^, ^35^, ^36^]

Short‐term exposure to xylene irritates the nose, eyes, and throat with subsequent harmful neurological, gastrointestinal, and reproductive effects. On the other hand, long‐term exposure to xylene can cause harmful effects on the respiratory, central nervous, cardiovascular, and renal systems.[^37^, ^38^, ^39^] According to the Occupational Safety and Health Act (OSHA), the permissible exposure limit (PEL), averaged over an 8 hour work shift, is 100 ppm and 200 ppm for xylene and toluene, respectively. Most exposures to xylene occur by inhalation or ingestion, after which this substance is readily absorbed leading to systemic toxicity. The vapor density of xylene is 3.8, therefore heavier than air, for which a value of 1 is considered. Such circumstances can cause asphyxiation in confined, poorly ventilated, or low‐ceilinged spaces. Although work environments can be appropriately adapted to reduce inhalation risks by installing local ventilation systems, and workers can be equipped with adequate personal protection equipment (PPE), the adoption of alternative solvents to xylene remains the best option to improve the environmental and safety of this kind of chemical process.^[40]^ When selecting alternative solvents, various aspects deserve special attention, including environmental impacts arising from the industrial production, recycling, and disposal processes, as well as EHS characteristics. Bio‐based solvents are gaining great attention as alternatives to fossil fuel‐based solvents. They can be produced from a range of feedstocks including vegetable oils, carbohydrates, citrus peels, lignin, and cellulose, to produce solvents such as biodiesel, glycerol, DMF, limonene, cyrene, etc.^[41]^ Recently, among non‐aromatic and bio‐based solvents, terpenes are finding many industrial applications as alternative extraction solvents capable of replacing organic solvents such as BTEX and chlorinated solvents.^[42]^ Limonene is an example of a bio‐based solvent, being produced from biomass or food waste. It belongs to the terpene class and is obtained in the two optical isomers, *d*‐limonene from citrus fruits and *l*‐limonene from lemongrass and citronella.^[43]^ The racemic mixture *dl*‐limonene, known also as dipentene, can be easily obtained by heating the pure enantiomers. Recently, from a circular economy perspective, the production of *dl*‐limonene by vacuum pyrolysis of used tires has been investigated.[^44^, ^45^]

Limonene has been reported as an innovative and versatile green chemical, increasingly used in different fields.^[46]^ It has a relatively high solvent power with a Kauri‐Butanol (KB) value around 60~100, which is comparable to the KB value typical of 98 for xylene. Recently, limonene has emerged as a green alternative to petroleum‐based solvents in chromatography or the extraction of natural products.[^47^, ^48^, ^49^, ^50^] Diverse industrial and household applications of citrus waste‐derived limonene as a solvent for cleaning, in substitution of BTEX, have been reported.^[51]^ Besides, due to its excellent affinity for lipophilic compounds, it has recently been tested for the bioremediation of contaminated petroleum.^[52]^ Moreover, limonene has been successfully employed as a co‐surfactant for the preparation of emulsions applied for the control of agricultural crop diseases^[53]^ and in phytomedicine.^[54]^ Also, limonene has been reported as a valuable sustainable solvent for depositing thin layers of molecular electronic materials such as solar cells,^[55]^ high‐performance polymer light‐emitting diodes (PLEDs), and optical field‐effect transistors (OFETs).^[55]^

Herein we describe the use of racemic limonene as an alternative (non‐toxic and bio‐based) solvent to xylene for the deposition of the thin layer containing carnauba wax as a release agent for the production of a peelable film.

## Results and Discussion

**Figure 2:**
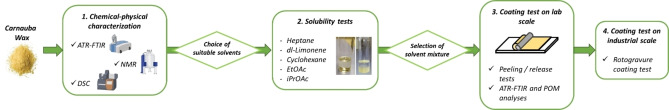
Schematic diagram illustrating the experimental workflow of the present study. This figure was partially created in BioRender. D'Orazio, G. (2024) BioRender.com/s59d816.

The experimental work in this study is outlined and illustrated in Figure 2. Carnauba wax samples underwent an initial deep structural characterization using ATR‐FTIR, NMR, and DSC. Based on the collected data, several solvents were selected and tested, including mixtures, to generate stable solutions of carnauba wax. After identifying the best solvent mixture in terms of solubility performance, sustainability, and toxicological profile, coating experiments were carried out on a laboratory scale. The results from these experiments were then used to set up and execute a coating test on an industrial scale.

### Chemical‐Physical Characterization of Carnauba Wax

The chemical composition of the carnauba wax samples used in the present study was investigated by combining information derived from NMR and ATR‐FTIR analyses.

**Figure 3:**
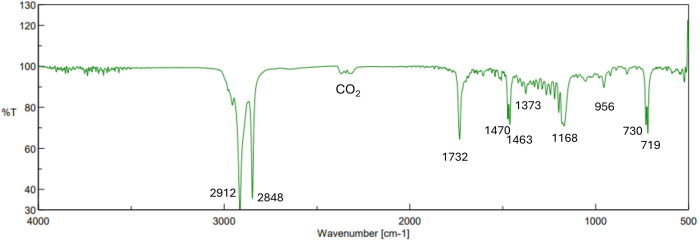
ATR‐FTIR spectrum of a Carnauba wax sample neat.

Specifically, as illustrated in Figure 3, the ATR‐FTIR spectrum of Carnauba wax showed two intense peaks at 2912 cm^−1^ and 2848 cm^−1^ ascribable to the ‐CH stretching of methyl and methylene groups, and two medium‐strong peaks at 1470 cm^−1^ and 1463 cm^−1^ due to methylene group (−CH_2_) in‐plane vibrations. A low prominent peak at 1373 cm^−1^ was attributed to the ‐CH_3_ bending of the aliphatic chains. Two medium‐strong peaks at 730 and 719 cm^−1^, due to the out‐of‐plane deformation of successive methylene groups (−(CH_2_)_n_), with n>4, are characteristic of long aliphatic chains and suggested the presence in Carnauba wax of long‐chain fatty acids, esters, and alkanes. Two intense absorption bands can be observed at 1732 cm^−1^ and 1168 cm^−1^ related to the presence of carbonyl groups of fatty acids and esters (−C=O), as an indication of the wax ester component. The very weak signal at 956 cm^−1^ was assigned to the −HC=CH− bending vibration ascribable to the characteristic di‐substituted trans‐olefin moiety of cinnamyl compounds.[^56^, ^57^]

**Figure 4:**
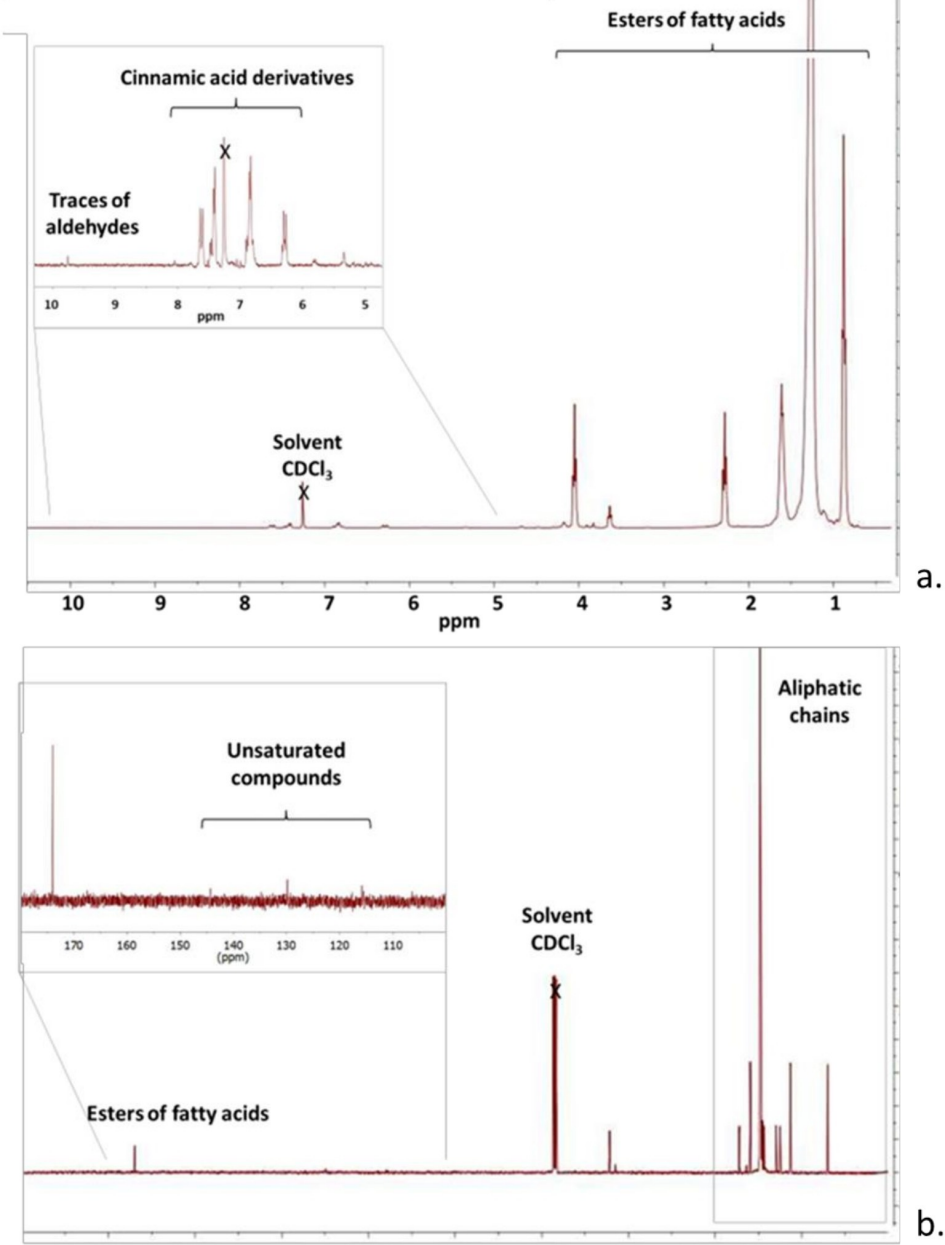
NMR spectra of a carnauba wax sample dissolved in CDCl_3_ were recorded at 305 K using a Bruker Avance 400 MHz spectrometer. a. ^1^H NMR spectrum. b. ^13^C{^1^H} NMR spectrum.

Additional details were obtained from the NMR measurements performed on samples of carnauba wax dissolved in deuterated chloroform according to the protocol described forehead. In the ^1^H NMR spectrum (Figure 4a), typical signals related to protons belonging to the saturated alkyl chains of fatty acid esters (FAEs) were identified. They include terminal methyl groups (−C**H_3_** at 0.88 ppm) and methylene groups (−(C**H_2_**)_n_‐ at 1.26 ppm), protons at α‐ and β‐position to the carbonyl group of FAEs (−C**H_2_**−C**H_2_**−COO−) in the spectral region from 1.15 to 1.72 ppm. Some relatively unshielded protons were identified at 3.64 and 4.31 ppm attributed to alkyl protons directly attached to the ester moiety (R‐COO‐C**H**_2_R).

The presence of a small number of unsaturated chains could be confirmed by the identification of very low signals attributed to the olefinic protons at 5.34 and 5.81 ppm (−C**H<C**=>C**H<C**‐>). Also, in the region 4.86 to 5.24 ppm, very low peaks were visible and were assigned to terpenic compounds, known to be commonly present in the carnauba wax.

Signals in the region 6.20–7.9 ppm indicated the presence of cinnamic acid derivatives. Furthermore, traces of aldehyde derivatives (RC**H**O) was detected as indicated by the presence of very low‐intensity signals at 9.74–9.90 ppm.

This chemical composition was confirmed by ^13^C{^1^H} NMR spectroscopy analyses (Figure 4b). Signals related to the methyl (−**C**H_3_) and methylene −(**C**H_2_)_n_‐ carbons of the aliphatic chains of FAEs were detected at 14.23 ppm and in the region 22.50–34.80 ppm, respectively. The signals at 64.54 ppm and 63.23 ppm were assigned to the carbons adjacent to the ester moieties. The quaternary carbon of ester groups (R**C**OOR) was detected at 174.15 ppm. Very low‐intensity signals were visible in the region 115–145 ppm, which were assigned to olefinic carbons contained in the unsaturated chains (−**C**H=**C**H−). The assignment of the compounds in the carnauba wax samples was confirmed by 2D NMR analyses (TOCSY and HMQC) and compared with the data reported in the literature.^[24]^

**Figure 5:**
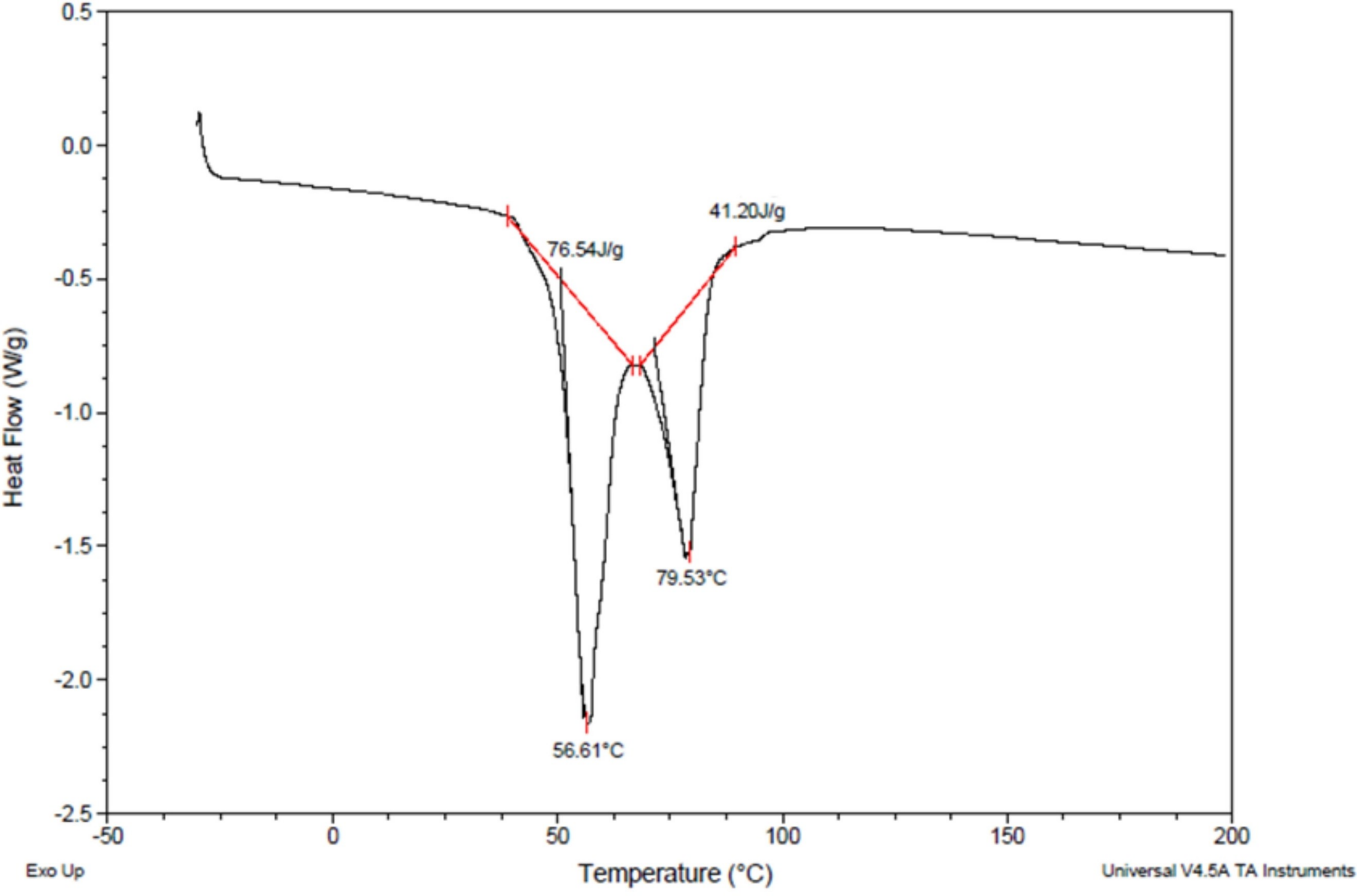
DSC analysis on Carnauba wax, carried out by using a TA Instrument differential scanning calorimeter.

Differential scanning calorimetry (DSC) was performed in a restricted temperature range to highlight the phase transition thermal event of the carnauba wax. As shown in Figure 5, two sharp endothermic transitions are distinguishable from each other at 56.61 °C and 79.53 °C, respectively, indicating a mixture of several molecular weights. A relatively high latent heat per unit volume (76.54 J/g and 41.20 J/g) characterized the analyzed samples. Based on data reported in the literature,[^24^, ^58^] the leading peak at 56.61 °C was attributed to a solid–solid phase transition (i. e. softening), while the peak events at 79.53 °C represented a solid‐liquid transition (i. e., melting).

### Solubility Tests

Solubility tests were conducted under controlled conditions to explore the feasibility of using alternative and more sustainable solvents in terms of health and safety properties than those currently used to dissolve carnauba wax before coating applications.

During the solubility tests, the contribution of the following parameters was evaluated: *i*. the percentage by weight of wax in the mixture; *ii*. the concomitant use of an additive with antistatic action; *iii*. the use of different solvents in variable ratios; *iv*. different thermal conditions. Determination of whether the sample of carnauba wax has dissolved was based entirely on visual observation. It was assumed that the chemical had dissolved if the solution was clear and showed no signs of cloudiness or precipitation. The stability of the obtained solutions was evaluated over 4 days at room temperature.

Based on the chemical characterization of carnauba wax resulting from the spectroscopic measurements (ATR‐FTIR and NMR), mixtures of solvents with a variable polarity profile were tested. Specifically, *dl*‐limonene and cyclohexane were tested as possible alternatives to xylene as a relatively less polar solvent, while *n*‐butylacetate, ethyl acetate, and isopropyl acetate were tested as solvents with a relatively higher polarity. Some crucial parameters were considered during the selection of the solvents, such as boiling point, flash point, autoignition temperature, and GHS hazard statements.

The solubility of carnauba wax in the prepared mixtures was evaluated by stirring them for 1 h at three different temperatures, namely room temperature, 40 °C, and 50 °C.

As described in Figure 5, different ratios of carnauba wax, additive, and solvents (in particular, different combinations of cyclohexane, isopropyl acetate (*i*PrOAc), ethyl acetate (EtOAc), and *dl*‐limonene) were examined. A cationic additive based on a compound solution of quaternary ammonium, coco alkyl dimethyl, ethyl sulfates has been tested as an antistatic agent to improve the resistivity features of the release layer. The tested ratios between carnauba wax and the additive were the following: a) wax 0.38 wt/vol % + additive 0.16 wt/vol %; b) wax 0.38 wt/vol %; c) wax 0.76 wt/vol % + additive 0.16 wt/vol %; d) wax 0.38 wt/vol % + additive 0.32 wt/vol %. The degree of solubility of the carnauba wax in the mixtures and the stability of the mixtures themselves over time were evaluated.

**Figure 6:**
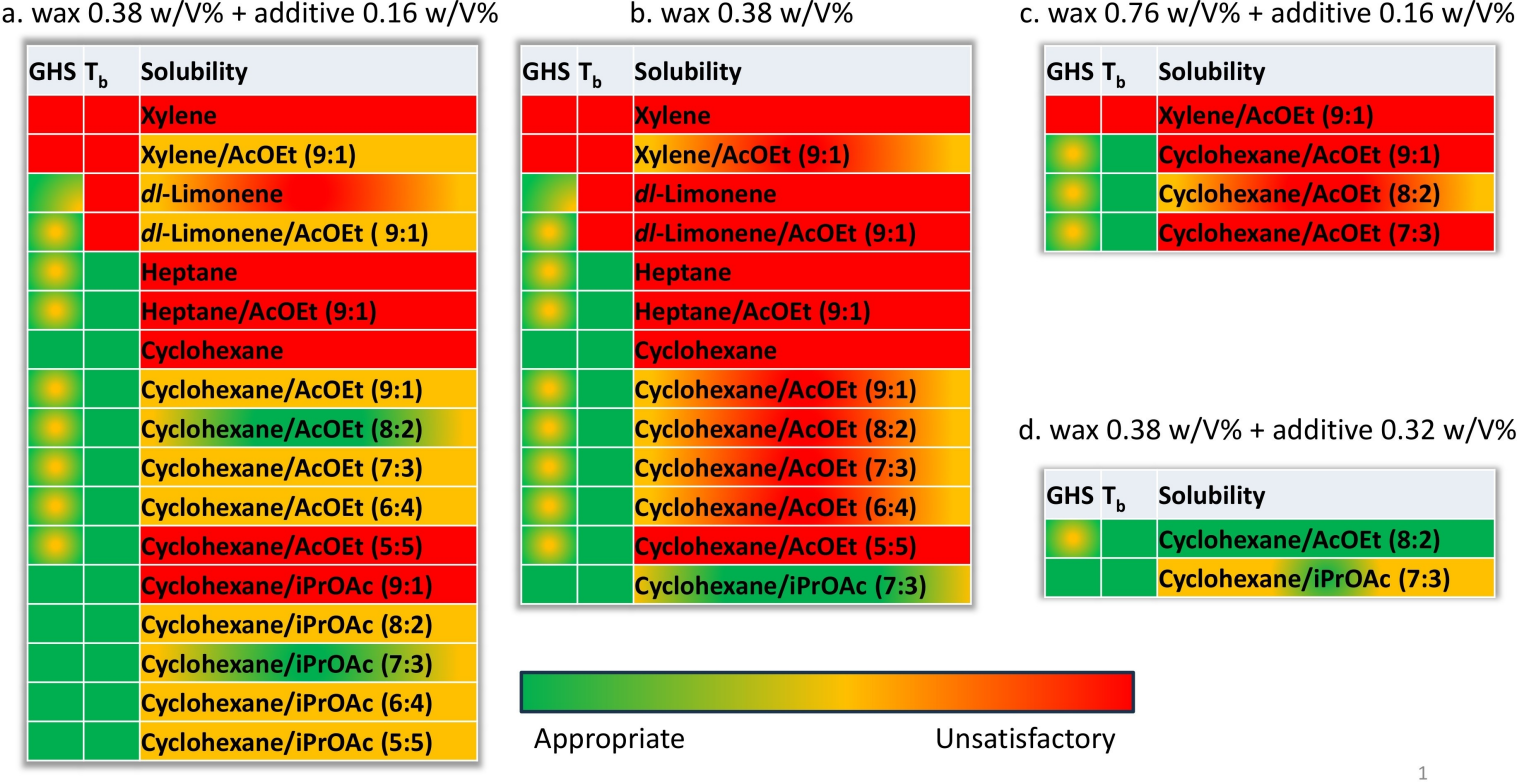
Suitability of carnauba wax‐based formulations in terms of solubility, boiling temperature (T_b_), and toxicological profile (GHS).

As summarized in Figure 6, the presence of the additive ensured better solubility and stability over time in the case of all the combinations tested, while a greater weight percentage of wax in the mixture (from 0.38 wt/vol % to 0.76 wt/vol %) had a decreasing effect on the solubility. The best results were obtained when cyclohexane was used in combination with EtOAc or *i*PrOAc in ratios of 8 : 2 and 7 : 3, respectively. It was observed that a higher amount of additive up to 0.38 wt/vol % under these conditions improved the solubility. It was observed that carnauba wax (0.38 wt/vol %) was sufficiently solubilized in a mixture of *dl*‐limonene and EtOAc in a ratio of 9 : 1 when 0.16 wt/vol % of additive was added to the mixture. The resulting mixtures showed good solubility after heating 1 h at 50 °C. Although these solutions showed instability over 4 days, the samples became as clear solutions again after a second cycling at 50 °C. The results obtained with these new combinations of solvents were very similar to those obtained with a commonly used mixture of xylene and EtOAc in a ratio of 9 : 1.

**Figure 7:**
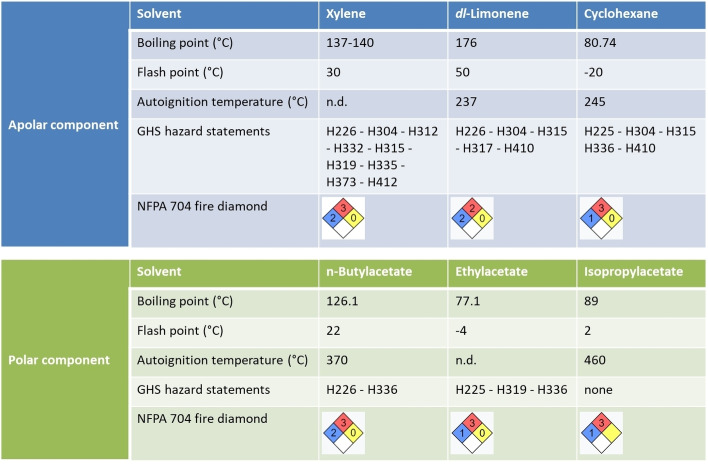
Health and safety evaluation of the most promising solvents according to the solubility tests. Data taken from the National Fire Protection Association (NFPA).^[59]^

The advantage of using *dl*‐limonene and cyclohexane as alternatives to xylene relies on the fact that they are characterized by a safer toxicological profile than xylene, as illustrated in Figure 7. However, compared to *dl*‐limonene, cyclohexane still shows an issue that makes its use problematic in an industrial process. Indeed, cyclohexane is characterized by a low flash point (−20 °C) and high vapor pressure (96.9 mmHg at 25 °C). Consequently, the risk of the fire hazard of cyclohexane is higher than *dl*‐limonene (flash point: 45 °C; vapor pressure: 1.55 mmHg) or xylene (flash point: 30 °C; vapor pressure: 8 mmHg). *dl*‐Limonene results as the safest solvent in terms of risk of fire hazard, being indexed as “2” and not “3” as for cyclohexane and xylene according to the National Fire Protection Association (NFPA).

Upon such considerations, the attention was focused on the release formulation obtained by dissolving carnauba wax and additive in a mixture of limonene and EtOAc according to the recipe illustrated in Table 1.

**Table 1:** Developed formulation of the release agent used to coat the PET foil.

**Chemical**	**Content**
*dl*‐Limonene	90 L
EtOAc	10 L
Carnauba wax	0.38 kg
Additive	0.16 kg

### Coating Tests on PET on a Laboratory Scale

The effectiveness as a release agent of the new formulation of carnauba wax in *dl*‐limonene and EtOAc was evaluated compared to that traditionally used in xylene and ethyl acetate (EtOAc).

The two release solutions were uniformly distributed over two separate sheets of PET by using a Mayer Rod Coater. A Mayer rod is a stainless steel rod tightly wrapped with stainless steel wires of a defined diameter. The rod scrapes off the excess coating solution and controls the weight per area of the coating; the thickness of the coating layer depends on the diameter of the wire.

The carnauba wax‐based release layer in the *dl*‐limonene/EtOAc mixture, once dried in an oven at 50 °C for 5 minutes, appeared transparent, smooth, and without creases. These results are comparable to those obtained with the classic formulation in xylene and ethyl acetate, with evident advantages in terms of eco‐toxicological profile.

Subsequently, an appropriate peelable layer was spread on two PET sheets, of which the first sheet had previously been coated with the new release formulation based on *dl*‐limonene/EtOAc and the second sheet coated with the classic release formulation based on xylene/EtOAc.

To evaluate the detachment capacity of the two formulations, a peeling test (FINAT Test Method no. 3^[60]^) was performed to determine the force required to separate the release backing from the pressure‐sensitive adhesive‐coated face material. A Release Testing Machine (ChemInstruments, AR‐1000) was used to peel the previously coated multilayer system through an angle of 180° with a jaw separation rate of 300 mm per minute. Five readings at 10 mm intervals from the center section of the test strip were taken. The average values of the five readings are listed in Table 2.

**Table 2:** Results of the peeling test. Low‐speed release force is expressed as the average result in Newton/5 cm width.

**Solvent Mixture**	**Release force (N/5 cm)**	**Standard deviation**
Xylene/EtOAc	0.25	0.06
*dl*‐Limonene/EtOAc	0.19	0.01

**Figure 8:**
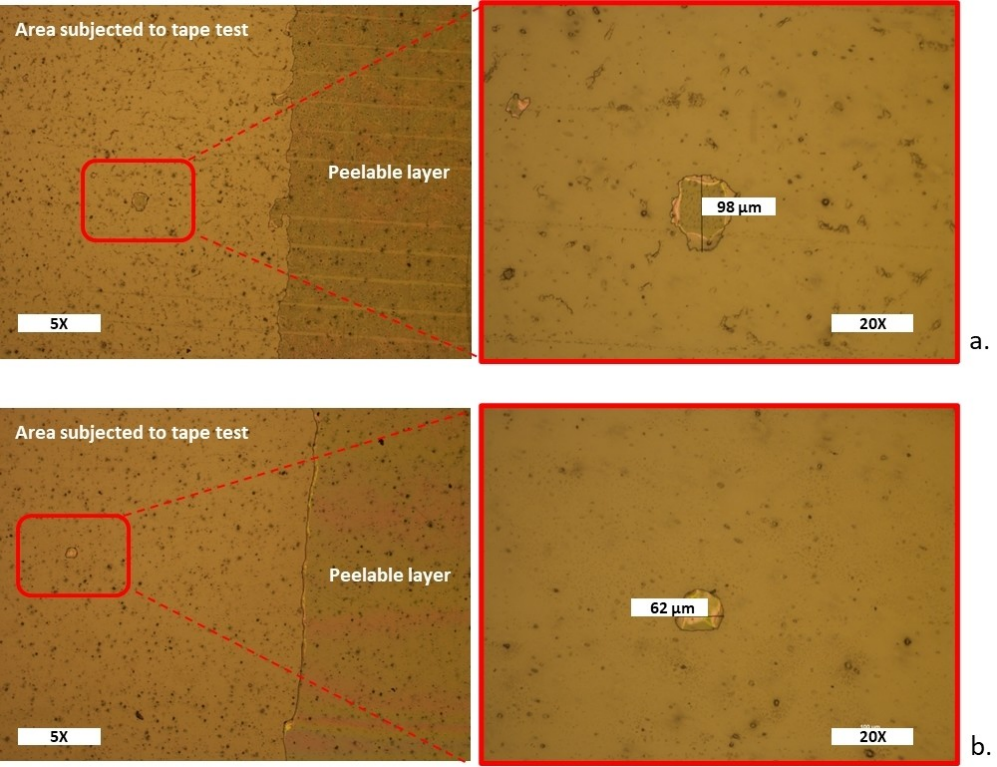
Polarized optical microscopy (magnification 5X e 20X) of PET sheets, which were spread with the release formulation containing carnauba wax dissolved in *dl*‐limonene/EtOAc (a) and xylene/EtOAc (b), coated with a peelable layer and, then, subjected to a conventional tape test.

Furthermore, ATR‐FTIR spectroscopy and polarized optical microscopy analyses were carried out to compare the releasing performance of the two formulations. As shown in Figure 8, the observation through the polarized optical microscope showed that, in both cases, the applied adhesive tape detached almost completely the overlying peelable layer producing few residuals on the surface. Interestingly, the residuals had a comparable size as using *dl*‐limonene as xylene (approximately≤100 micrometers).

**Figure 9:**
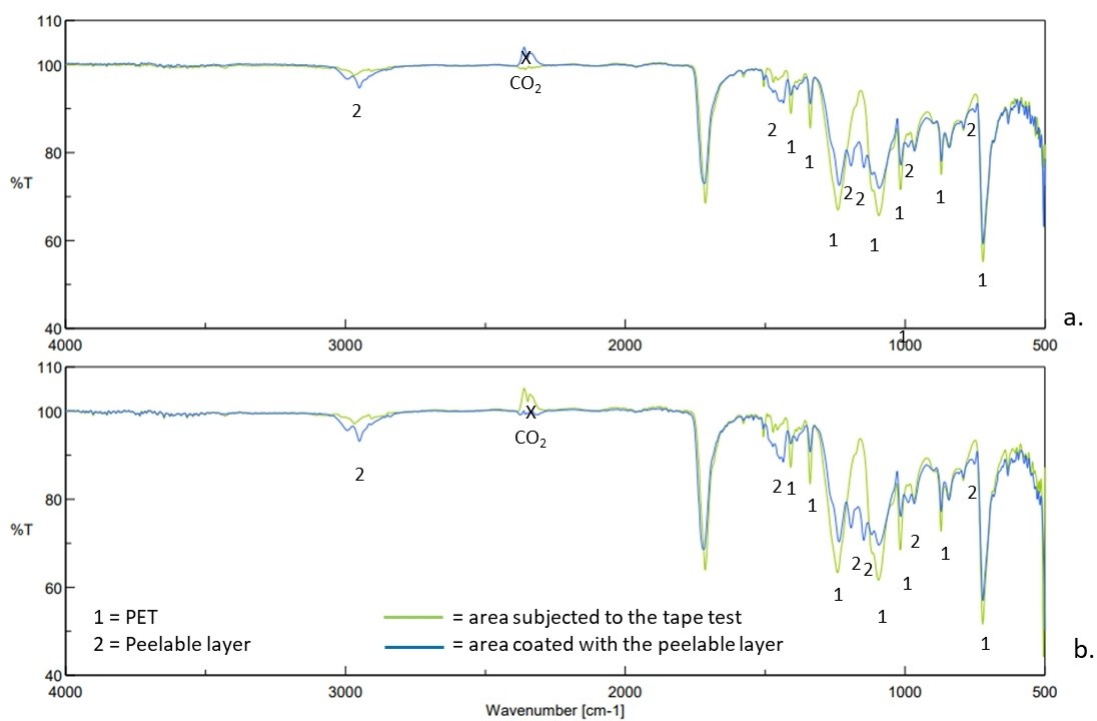
ATR‐FTIR analyses of PET sheets, which were spread with the release formulation containing carnauba wax dissolved in *dl*‐limonene/EtOAc (a) and xylene/EtOAc (b), coated with a peelable layer and, then, subjected to a conventional tape test.

The ATR‐FTIR measurements were performed both on the area subjected to the tape test and on the area containing the double layer consisting of the release layer and the peelable layer previously deposited in succession. The typical signals of the PET were identified in the ATR‐FTIR spectrum of the areas that were subjected to the tape test, demonstrating that a successful removal of the peelable layer occurred. As confirmation, such peaks attributed to the PET were not detected in the ATR‐FTIR spectrum of the adjacent area, where the peelable layer was still adherent, thus, covering the PET layer. As shown in Figure 9, similar results were obtained when the two formulations of release were compared.

### Coating Tests on PET on an Industrial Scale

**Figure 10:**
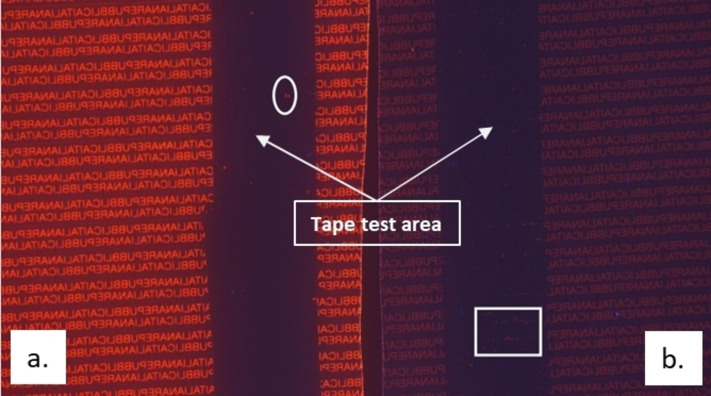
Ultraviolet‐visible spectroscopy of PET sheets, which were spread through an industrial rotogravure machine with the release formulation containing carnauba wax dissolved in *dl*‐limonene/EtOAc/additive (a) and xylene/EtOAc/additive (b), coated with a UVA‐365 absorbing peelable layer and, then, subjected to a conventional tape test.

Subsequently, two separate coating tests on an industrial scale were carried out on PET support using a rotogravure machine, to compare the release efficiency of the formulation of carnauba wax solubilized in xylene/EtOAc and *dl*‐limonene/EtOAc, respectively. Following a drying step, a layer containing a peelable UVA‐365 absorbing film was deposited on the first layer containing the release formulation. As illustrated in Figure 9, after a tape test, small‐sized residues were detected in the area subjected to the tape removal, confirming that the performance of the formulation based on *dl*‐limonene/EtOAc/additive is comparable to that one based on xylene/EtOAc/additive. Such a result is very promising for the use of *dl*‐limonene as a more sustainable alternative to xylenes in this kind of production process (Figure 10).

## Conclusions

In conclusion, this study focused on the search for more sustainable alternative solvents to be used for the industrial‐scale preparation of release formulations for use in the production of peelable foils. In this context AH solvents, such as toluene and xylenes, are the most commonly used solvents, with intrinsic critical issues in terms of health and safety, according to the International Agency for Cancer Research (IARC), the Occupational Safety and Health Act (OSHA), and the National Fire Protection Association (NFPA). Considering the large volumes used during an industrial production process, it is urgent to improve the sustainability of the solvents used as soon as possible, preserving the efficiency of the process and the quality of the finished product. Following the chemical‐physical characterization of the carnauba wax adopted as a release agent, an in‐depth study was carried out to identify the most suitable mixture of solvents to obtain a clear and stable solution over time. The *dl*‐limonene/EtOAc mixture in the 9 : 1 ratio was identified as the most suitable one to completely solubilize the carnauba wax (0.38 wt/vol %) and obtain a stable solution over time. The effect of adding a quaternary ammonium derivative to the formulation as an additive with antistatic properties was also evaluated. It has been found that it can facilitate the application process and improve the quality of the coated product in terms of transparency and smoothness. The developed formulation was tested in an industrial process providing results comparable to the formulation currently used which is based on carnauba wax solubilized in AH solvents. The use of *dl*‐limonene as an alternative to xylene will improve workplace health and safety conditions, with a reduction in toxicological concerns and fire risks along the production line.

## Experimental Section

### Materials and Methods

#### Materials

All solvents used were ACS grade or reagent grade. Deuterated chloroform (≥99.8 atom % D, CAS N. 865–49–6), cyclohexane (99.5 %, CAS N. 110–82–7), iPrOAc (99.6 %, CAS N. 108–21–4), EtOAc (99.6 %, CAS N. 141–78–6), xylene (xylenes, reagent grade, CAS N. 1330–20–7), heptane (≥99 %, CAS N. 142–82–5) were purchased from Sigma Aldrich (Milan, Italy); *dl*‐limonene (mixture of *d*‐ and *l*‐form ≈1 : 1, CAS N. 138–86–3) was purchased from EMD Millipore (Burlington, Massachusetts, United States). Adhesive tape for tape test (04104–00015–00, 66 m×19 mm, 3/4”:72 yds) was purchased from Tesa SE (Norderstedt, Germany).

#### Solubility Tests

Table 3 lists the solution groups adopted during the solubility tests, considering the different wax concentrations, the presence or absence of the additive, and the different solvent mixtures (cyclohexane, *i*PrOAc, EtOAc, xylene, heptane, and *dl*‐limonene) adopted.

**Table 3:** List of the solution groups tested during the solubility tests.

**Solution group a: wax 0.38 wt/vol % + additive 0.16 wt/vol %**		
Solvent 1	Solvent 2	Solvent 1/Solvent 2 ratio
xylene	‐	10:0
xylene	EtOAc	9 : 1
*dl*‐limonene	‐	10:0
*dl*‐limonene	EtOAc	9 : 1
heptane	‐	10:0
heptane	EtOAc	9 : 1
cyclohexane	‐	10:0
cyclohexane	EtOAc	9 : 1
cyclohexane	EtOAc	8 : 2
cyclohexane	EtOAc	7 : 3
cyclohexane	EtOAc	6 : 4
cyclohexane	EtOAc	5 : 5
cyclohexane	*i*PrOAc	9 : 1
cyclohexane	*i*PrOAc	8 : 2
cyclohexane	*i*PrOAc	7 : 3
cyclohexane	*i*PrOAc	6 : 4
cyclohexane	*i*PrOAc	5 : 5

Solution group b: wax 0.38 wt/vol %		
Solvent 1	Solvent 2	Solvent 1/Solvent 2 ratio
xylene	‐	10:0
xylene	EtOAc	9 : 1
*dl*‐limonene	‐	10:0
*dl*‐limonene	EtOAc	9 : 1
heptane	‐	10:0
heptane	EtOAc	9 : 1
cyclohexane	‐	10:0
cyclohexane	EtOAc	9 : 1
cyclohexane	EtOAc	8 : 2
cyclohexane	EtOAc	7 : 3
cyclohexane	EtOAc	6 : 4
cyclohexane	EtOAc	5 : 5
cyclohexane	EtOAc	7 : 3

Solution group c: wax 0.76 wt/vol % + additive 0.16 wt/vol %		
Solvent 1	Solvent 2	Solvent 1/Solvent 2 ratio
xylene	EtOAc	9 : 1
cyclohexane	EtOAc	9 : 1
cyclohexane	EtOAc	8 : 2
cyclohexane	EtOAc	7 : 3

Solution group d: wax 0.38 wt/vol % + additive 0.32 wt/vol %		
Solvent 1	Solvent 2	Solvent 1/Solvent 2 ratio
cyclohexane	EtOAc	8 : 2
cyclohexane	EtOAc	7 : 3

The solubility of the obtained mixtures was tested by stirring them for 1 h at three different temperatures, i. e. *i*. room temperature, *ii*. 40 °C, and *iii*. 50 °C. Determination of whether the sample of carnauba wax has dissolved was based entirely on visual observation. It was assumed that the chemical had dissolved if the solution was clear and showed no signs of cloudiness or precipitation. The stability of the obtained solutions was evaluated over 4 days at room temperature.

#### Spreading Tests

Mayer Rod Coater (K303S Multi Coater ‐ RK Print Coat Instruments) was used to perform surface coating on a laboratory spreading scale. A small volume of each release formulation was deposited (by a 1 mL Pasteur pipette) on the top of a foil of PET (20×40 cm), then the wired wound rod (Mayer rod number 1, spreading rate=3 mt/min) was used to doctor the excess coating solution and control the coating weight. The final wet thickness after doctoring was 6 μm. Complete evaporation of the solvent was obtained by keeping the coated sheet in a hoven (ArgoLAB TCF 200 plus) for 5 minutes at 50 °C.

#### Peeling Adhesion 180° Tests

A Release Testing Machine (ChemInstruments, AR‐1000) capable of peeling a laminate through an angle of 180° with a jaw separation rate of 300 mm per minute and accuracy of 2 % was used. Five strips (50 mm wide and a minimum length of 175 mm in the machine direction) were obtained from each representative sample. The strips were adhered to a test plate with double‐sided tape and kept between two flat metal plates for 20 hours at 23 °C under a pressure of 6.86 kPs to ensure good contact between the release material and the adhesive. The strips were removed from between the plates and kept for not less than 4 hours at 23 °C, 50 % RH. The laminate was peeled apart at an angle of 180° with a jaw separation speed of 300 mm per minute. The average peeling force (centinewtons per 50 mm width of the sample) was recorded from the center section of the test strip from at least 5 readings at 10 mm intervals.

#### ATR‐FTIR Analysis

The infrared spectroscopy measurements were carried out in transmittance mode on carnauba wax samples with attenuated total reflectance (ATR) employing a JASCO FTIR 4200 Spectrometer equipped with a ZnSe cell window (JASCO Co., Tokyo, Japan). Infrared spectra were recorded in the spectral range 4000 to 500 cm^−1^, resolution 4 cm^−1^, accumulation 50 times, and 96 scans.

The raw data were processed using Spectra Manager software. The most representative bands were assigned based on the literature and reference spectra.

#### NMR Spectroscopy

NMR spectra were recorded through a Bruker Avance 400 MHz spectrometer equipped with a 5 mm inverse probe. The pulse lengths were calibrated before each experiment, and the probe tuning and matching were adjusted for each sample. The temperature of the probe was set at 305 K. An amount of 30 mg of carnauba wax was dissolved in 600 μL of deuterated chloroform (CDCl_3_). The resulting solution was transferred to an NMR tube (Norell 509‐UP 7, Norell, Landisville NJ, United States).

The following acquisition parameters were used to record ^1^H NMR spectra: pulse program=zg; size of fid (TD)=64 K; spectral width (SW)=20 ppm; transmitter off‐set=5.00 ppm; dummy scans (ds)=0; number of scans (ns)=4; acquisition time=4.09 s; recycle delay (d1)=10.0 s. The following acquisition parameters were used to record ^13^C{^1^H} NMR: pulse program=zgdc; size of fid (TD)=128 K; spectral width (SW)=200 ppm; transmitter offset=100 ppm; dummy scans (ds)=0; number of scans (ns)=4848; acquisition time=3.26 s; recycle delay (d1)=5 s.

NMR raw data (Free Induction Decays, FIDs) were processed using the software MestReNova 14.3.3 software (Mestrelab Research SL, Santiago de Compostela, Spain). The phase and the baseline were optimized manually. The zero‐order (PH0) and first‐order (PH1) phase parameters were adjusted opportunely after having set the pivot parameter to the biggest peak in the spectrum. The baseline correction was applied to flatten the baseline on the Fourier Transformed data. The Multipoint Baseline Correction was adopted, upon selecting opportunely the points corresponding to baseline regions (also known as control points) which were then used by the software to build a baseline model using the interpolation algorithm. The chemical shifts are reported in ppm and referenced to the solvent residual singlet peak at δ=7.26 in ^1^H NMR spectra and the triplet peak relating to the chloroform‐*d* centered at δ=77.16 in ^13^C{^1^H} NMR spectra, respectively, were used as chemical shift references.

#### Differential Scanning Calorimetry (DSC)

For the differential scanning calorimetry (DSC) analysis, a quantity of about 2.0 mg carnauba wax was weighed in an aluminum pan and placed into the thermal analyses chamber (DSC Q2000, TA Instrument, France). For each measurement, an empty aluminum pan was used as a reference. Via a personal computer and separate thermal analysis processor (TA Instruments Universal Analysis 2000) the following conditions were set: start at 50 °C, rate of heating 20 °C per minute, and final temperature of 300 °C. The following parameters were measured: melting temperature (T_m_, °C, taken as the peak temperature of the largest endothermic event), the onsets and offsets of melting (T_on_ and T_off_, respectively), and the characteristic thermal properties (enthalpy of melting, ΔH_m_, (J/g).

## Author Contributions

Conceptualization, B.M., and V.G.; methodology, B.M., G.D, and R.R.; validation, B.M., G.D., A.R., and F.S.A.; formal analysis, F.S.A., G.D., R.R., A.C.; writing—original draft preparation, B.M., and R.R.; writing—review and editing, B.M., V.G., M.L, P.M., and A.R.; visualization, B.M., G.D., and M.C.; supervision, V.G. and B.M.; project administration, V.G., P.M., M.L., M.C., and G.G.; funding acquisition, V.G., M.L., P.M., and G.G. All authors contributed to the preparation of the manuscript.

## Conflict of Interests

The authors declare no conflict of interest.

1

## Data Availability

The data that support the findings of this study are not publicly available due to their containing information that could compromise the privacy of research participants.
